# Supplementary figures and images for: Perceptions toward Ebola vaccination and correlates of vaccine uptake among high-risk community members in North Kivu, Democratic Republic of the Congo
Source: PLOS Glob Public Health. 2024 Jan 18;4(1):e0002566. doi: 10.1371/journal.pgph.0002566 (PMC10796044; doi:10.1371/journal.pgph.0002566)

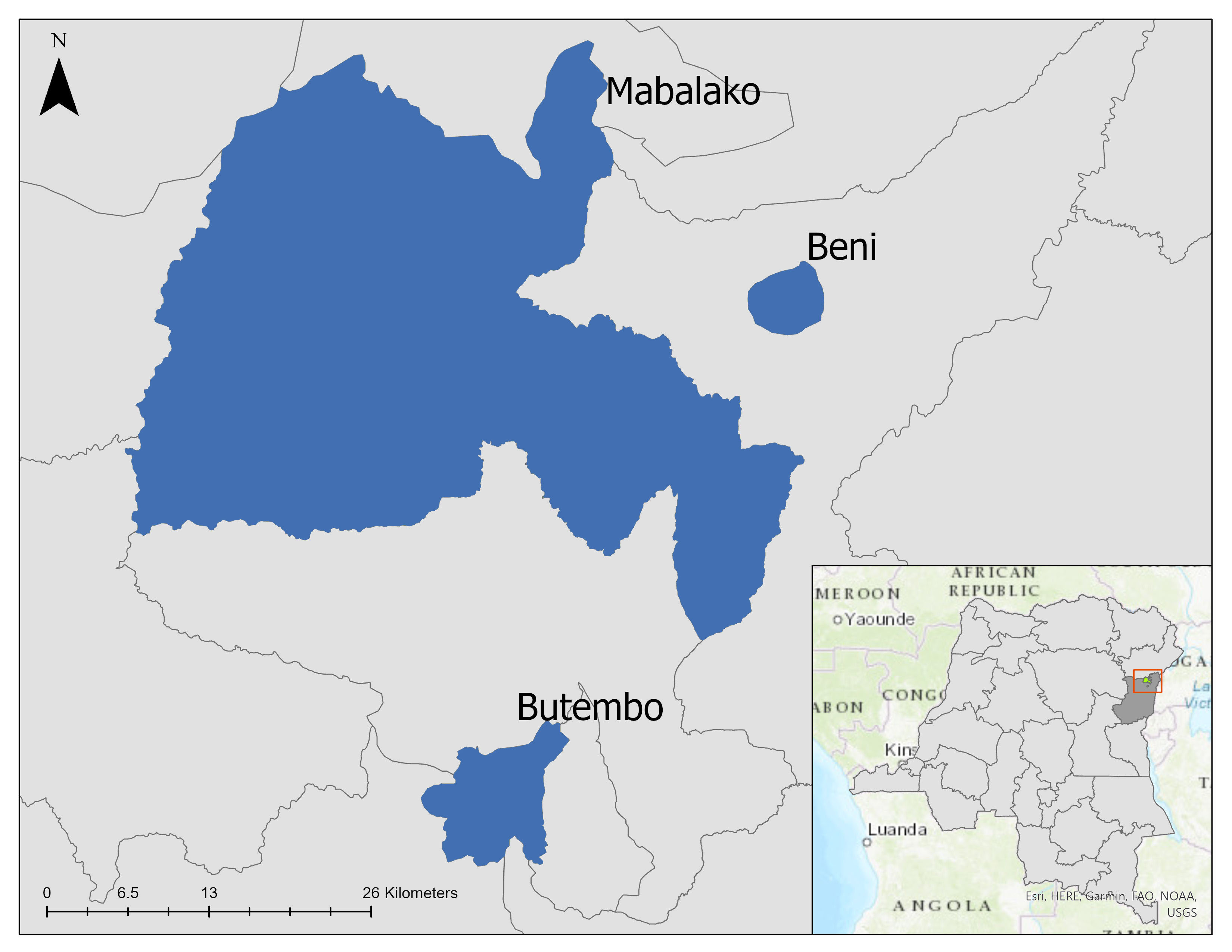

Supplement: S1 Fig — Source: https://data.humdata.org/dataset/rdc-statistiques-des-populations. (TIFF) [file pgph.0002566.s001.tiff]
