# Supplementary material for: Perceptions toward Ebola vaccination and correlates of vaccine uptake among high-risk community members in North Kivu, Democratic Republic of the Congo
Source: PLOS Glob Public Health. 2024 Jan 18;4(1):e0002566. doi: 10.1371/journal.pgph.0002566 (PMC10796044; doi:10.1371/journal.pgph.0002566)
Supplement: S1 Table — (DOCX) [file pgph.0002566.s002.docx]

**S1 Table.** Perceptions towards routine immunizations among community members, North Kivu, Democratic Republic of the Congo, 2021

|  | **Very much** | **Somewhat** | **Very Little** | **Not At All** | **Don’t Know/**  **Declined** |
| --- | --- | --- | --- | --- | --- |
| Questionnaire Item | **n(%)**  **N=631** | | | | |
| How much do you think that vaccines are good? | 240 (38.0) | 220 (34.9) | 72 (11.4) | 40 (6.3) | 59 (9.4) |
| How much do you think that vaccines are safe? | 170 (26.9) | 209 (33.1) | 33 (21.1) | 54 (8.6) | 65 (10.3) |
| How much do you think that vaccines protect against diseases? | 255 (40.4) | 233 (36.9) | 77 (12.2) | 25 (4.0) | 41 (6.5) |
| To which extent do religious leaders in your community approve of vaccination | 314 (49.8) | 154 (24.4) | 50 (7.9) | 14 (2.2) | 99 (15.7) |
| How much do other leaders in this community approve of vaccination? | 290 (46.0) | 186 (29.5) | 42 (6.7) | 17 (2.7) | 96 (15.2) |
|  | **Positively** | **Mixed** | **Negatively** | **-** | **Don’t Know/**  **Declined** |
| How do people in this community usually speak about vaccination? | 84 (13.3) | 354 (56.1) | 136 (21.6) | - | 57 (9.0) |
