# Supplementary material for: Perceptions toward Ebola vaccination and correlates of vaccine uptake among high-risk community members in North Kivu, Democratic Republic of the Congo
Source: PLOS Glob Public Health. 2024 Jan 18;4(1):e0002566. doi: 10.1371/journal.pgph.0002566 (PMC10796044; doi:10.1371/journal.pgph.0002566)
Supplement: Attachment — Submitted filename: Response to Reviewers_10-13-23.docx [file pgph.0002566.s004.docx]

Thank you very much for your decision letter and for the excellent and constructive reviewer comments. Please find below our detailed responses to reviewer comments. We have attached a “tracked changes” version which includes the updates and a final clean version of the manuscript. On behalf of my co-authors, we thank you very much for your consideration of our revised manuscript for publication. Please do not hesitate to contact us with any additional changes that might be required.

**Reviewer #1**

Using a cross-sectional survey, the authors of this piece assess community perceptions towards Ebola vaccination among high-risk individuals who were likely offered an Ebola vaccine in North Kivu, Democratic Republic of the Congo (DRC), during the DRC's 10th Ebola outbreak. They use the survey results to run a multivariate regression that measures associations between vaccination uptake and several covariates. This study is useful in understanding vaccination patterns in this community, and may have useful implications for future vaccination efforts. I have several comments that I would like the authors to address:

**Major**

1. **Lines 71-72: The authors say here that there is a strong distrust of foreigners and government in this community. How did the authors deal with this? Did they use local colleagues who were non-government-affiliated to do the surveys? This would be good to specify, as it otherwise leaves room for substantial bias in the results.**

International Medical Corps recruited local data collectors who were non-government- affiliated and could speak the predominant local language(s). These details have been added to Lines 106-110.

2. **Line 75: how was “high risk for contracting EVD” defined?**

We have added more details to make this clearer and defined those who were likely eligible for ring vaccination in Lines 77-81.

3. **Table 2: There were a sizable number of “Other” responses for “If you heard negative information, what information did you hear?”. It would be interesting to delve into what some of those were.**

Thank you for your suggestion. We have included an additional category “The vaccine will kill us” to the table, separating it from the “Other” responses. For all other responses where there were fewer than 10 responses, the following details were added to the text: “Other negative information respondents heard was that the vaccine is experimental, contaminated, and not accepted by religious leaders. A few respondents reported hearing that healthcare personnel receive a different vaccine than the rest of the population.” These details have been added to Lines 187-193.

4. **Table 3: It’s an interesting discrepancy that the strong majority of people believe that the vaccine is needed to prevent disease spread during an outbreak, but that 20% of people disagree that vaccination prevents EVD. It would be interesting to discuss that further, space permitting. Similarly, the result that 30.6% reported not wanting to be vaccinated when the vaccine was available conflicts with the percentage that did get vaccinated, and this would likewise be interesting to comment on**

We thank the reviewer for their suggestions, and these excellent observations. Regarding the first point, this apparent discrepancy may be explained by respondents understanding that vaccination is not 100% effective, although it can reduce community transmission. This is seen with many other vaccine preventable diseases such as COVID-19, where vaccination is not 100% effective, but very helpful in reducing severity. It is also possible that this discrepancy is attributed to knowledge of breakthrough infections, which did occur, especially among contacts who were vaccinated during their incubation period. Additionally, further explanations will be explored in the qualitative component of this work, which was done in conjunction with this survey.

Regarding the second point, while the great majority of respondents did receive the vaccine, the 30% who had reservations regarding vaccination may have been eventually convinced to be vaccinated due to improved understanding of the safety/efficacy of the vaccine over time, as well as additional drivers such as social desirability (from repeated targeted campaigns to encourage vaccination in the communities), and potentially from fear of vaccination teams if they did not accept vaccination. These issues would also be better addressed using qualitative methods which are planned.

We have added information on these points to the discussion section.

5. **Correlates of vaccine uptake/Table 6: Did authors test for collinearity in their regression? There are several covariates that I would expect to be highly correlated (e.g., heard negative information about the vaccine and ebola vaccine has severe side effects).**

Thank you for this comment, we clarify that we did examine collinearity using variance inflation factors (VIF) for the regression analysis, as well as correlation between candidate variables considered for the regression analysis. Variables with strong collinearity (r>0.8) were excluded. VIF for the model was 4.8, indicating an acceptable level of multicollinearity. Additionally, the variable selection was primarily driven by current theoretical understanding of drivers of vaccine hesitancy - certain variables such as hearing negative information about vaccines were thought to reflect general levels of community distrust of Ebola vaccines, while the question on belief the vaccine causes side effects reflects individual perception of dangers of vaccination therefore these variables were both included as they assess different potential drivers.

6. **Discussion: In the limitations section, it would be important for the authors to comment on how their results may be affected by the fact that the participants, at the time of study, had lived through an Ebola outbreak. When the outbreak was beginning (and thus when they were offered the vaccinations), their perceptions around vaccinations and disease severity may have been quite different. This could have important implications for new outbreaks when vaccinations are being offered.**

Thank you for the suggestion, we agree and have revised the discussion Lines 380-394 to include this point.

7. **Lines 286-287: The authors comment that a two-dose vaccine regime was offered in areas near the active outbreak. Was North Kivu included in this? If so, are the study results taking into account differences between individuals who got one vaccination versus both?**

Thank you for the comment. Yes, this took place in North Kivu, but not in the health zones where the outbreak was occurring. It is highly unlikely that our population received the other vaccine. We have revised the discussion Lines 318-3319 to be clearer.

8. **In the Discussion, the authors mention and cite several sources that appear to have done similar work in North Kivu. How does this study differentiate itself?**
   Thank you for your comment. Our study was different in that unlike the other studies that mostly surveyed affected communities earlier in the outbreak, our survey assessed perceptions toward the Ebola vaccination at the end of the outbreak in areas that were frequent Ebola epicenters. Also, our survey is one of the few that has investigated outbreak experiences of respondents, positive and negative information heard about the Ebola vaccine, and general vaccine confidence. We have added these details to the Discussion section.

**Minor**

9. **Line 38: Remove extraneous )**

Thank you, we have deleted this.

10. **Lines 56-58: Is there a reference for this?**

Thank you for pointing this out, we have re-added the references to these lines.

11. **Throughout: make sure Ebola versus EVD use is consistent.**

We thank the reviewer for this suggestion and have made sure to be more consistent with the terms Ebola versus EVD. When referring to the vaccine or the virus, we use “Ebola” and for all other Ebola references we use “EVD”.

12. **I suggest reorganizing Table 2 so that the answers for “If you heard positive/negative information, what information did you hear?” are in order based on response percentage**

Thank you for your suggestion, we have reordered the responses based on percentage.

13. **Line 249: Please define RCCE at first use**

Thank you, we have placed an abbreviation where it was first mentioned in the Introduction (Line 28).

14. **Line 293-294: What is the timeframe for this?**

Lines 293-94 are referring to the 10^th^ EVD outbreak. We have added the timeframe “2018-2020” to clarify.

15. **How does this study differentiate between prior studies in North Kivu that authors mention?**

Thank you for your comment. Our study was different in that unlike the other studies that mostly surveyed affected communities earlier in the outbreak, our survey assessed perceptions toward the Ebola vaccination at the end of the outbreak in areas that were frequent Ebola epicenters. As a result, our respondents, who were at high-risk for EVD infection, had prior experience with Ebola and were heavily targeted during the course of the outbreak by Ebola vaccination campaigns. Also, our survey is one of the few that has investigated outbreak experiences of respondents, positive and negative information heard about the Ebola vaccine, and general vaccine confidence. We have added these details to the Discussion section.

16. **Table S2: Caption says “eligible but unvaccinated,” but it appears as though all participants are included here**

Thank you for pointing this out, we have corrected the caption to “Perceptions towards routine immunizations among community members, North Kivu, Democratic Republic of the Congo, 2021.”

17. **Table 3 and S2: I suggest switching out the rows currently included in Table 3 for Attitudes on Vaccines in General with some of those included in Table S2. The responses for "How much do you think that vaccines are good?,” “How much do you think that vaccines are safe?,” and "How much do you think that vaccines protect against diseases?” from Table S2 are particularly interesting, and including them in Table 3 instead of the current rows would allow for comparisons between results for Ebola vaccination and general vaccination.**

Thank you for your suggestion, unfortunately since the responses in Table 3 and S3 were graded on different Likert scales (ex: 5 ratings in Table 3 versus 4 ratings in S2), we feel it would not be appropriate to merge them into the same table.

**Reviewer #2**

**The article has a clear description of the survey setting, study design, data collection, and analysis methods. There are a few suggestions and comments that could enhance the clarity and completeness of the article in general.**

1. **One is to provide more information about why the specific health zones were chosen based on their high case counts, so it would be easier to understand the rationale behind the selection.**

The health zones (Beni, Butembo, and Mabalako) were selected because of their high case counts and persistent community resistance to response activities (Lines 79-81). Additionally, these locations were selected due to IMC’s operational presence and were therefore chosen based on accessibility.

2. **Other recommendation is to explain more about the "ring vaccination" concept in a bit more detail, as it might not be familiar to all readers.**

Thank you for your recommendation. We have added more detail about the ring vaccination approach in the Introduction Lines 36-42.

3. **When describing the sample size calculation, what was the rationale behind the chosen values for parameters like the design effect (DEFF) and the intracluster correlation.**

Given the unique design of this study which used a sampling approach which mimicked ring vaccination, there are no standardized DEFF or ICC values based on prior research for which to rely on. However, the approach was deemed to be most similar to other vaccination coverage cluster surveys as well as a similar study used in a cross-sectional household vaccination survey in Sierra Leone from which we used these DEFF and ICC values for purpose of sample size calculation.

4. **Do the 39 Ebola survivors that were randomly selected served as focal points for recruitment or as a point of reference for the clusters?**

The 39 Ebola survivors served as the point of reference for the clusters. Survivors, their household members, and members of survivors’ neighboring households were eligible for inclusion. We have changed Line 90-91 to “The local voluntary community EVD survivors’ association provided a list of all EVD survivors, from which 39 persons were randomly selected as a point of reference for the clusters.”

5. **Detail the process of translation from French to Swahili by data collectors. This process could introduce translation bias or issues, and it's important to discuss how this was mitigated.**

Thank you, we have added additional details. Data collectors translated to Swahili on an ad hoc basis based on participant preferences. Our local data collectors were chosen due to their fluency in both languages. Translation from French to Swahili was practiced by data collectors during the training.

6. **In the data analysis section, consider expanding on the choice of the modified Poisson regression model using 'xtgee' in STATA for analyzing associations. Explain why this method is suitable for the data and research questions.**

Thank you for this comment. We have expanded upon the reasons for selecting modified Poisson regression, an increasingly used alternative to binomial regression for analysis of clustered data. We have added the following to Lines 130-33, “Modified Poisson regression has been suggested as a preferable alternative to binomial regression due to easier interpretability of relative risks (versus odds ratios) and due to improved approximation of risk when the outcome is not rare.”

7. **Consider providing a bit more context on the rationale behind choosing specific explanatory variables for inclusion in the regression model. This would help readers understand the factors that were deemed important in relation to vaccine uptake.**

Thank you, we have added additional details to the Methods section Lines 138- 145. We used current theoretical understanding (models such as 3C, 5C, and health belief models) of key drivers of vaccine hesitancy to develop our model. Current understanding of vaccine hesitancy suggests factors such as beliefs regarding vaccine safety/efficacy, perception of risk, desire to protect oneself and ones’ community, as well as trust and confidence in the vaccine are key drivers of vaccine hesitancy.
